# Characteristics of the Accessible Chromatin Landscape and Transcriptome under Different Temperature Stresses in *Bemisia tabaci*

**DOI:** 10.3390/genes14101978

**Published:** 2023-10-23

**Authors:** Xiaona Shen, Xiaodi Wang, Nianwan Yang, Fanghao Wan, Zhichuang Lü, Jianying Guo, Wanxue Liu

**Affiliations:** 1 State Key Laboratory for Biology of Plant Diseases and Insect Pests, Institute of Plant Protection, Chinese Academy of Agricultural Sciences, Beijing 100193, China; naxiaoshen@163.com (X.S.); 82101195160@caas.cn (X.W.); yangnianwan@caas.cn (N.Y.); wanfanghao@caas.cn (F.W.); guojianying@caas.cn (J.G.); liuwanxue@caas.cn (W.L.); 2 Department of Basic Medicine, Changzhi Medical College, Changzhi 046000, China; 3 Institute of Western Agriculture, The Chinese Academy of Agricultural Sciences, Changji 831100, China

**Keywords:** temperature stress, chromatin accessibility, tolerance, epigenetic regulation

## Abstract

*Bemisia tabaci* is an important invasive pest with worldwide distribution and strong temperature tolerance. Previous studies have shown that temperature tolerance varies significantly between the different invasive populations. Several key factors involved in epigenetic regulation have been identified and verified in *B. tabaci*; therefore, epigenetic adaptation mechanisms may also exist. This study aimed to detect changes in the chromatin accessibility landscape and genome-wide transcriptome under different temperature stresses in *B. tabaci*. Assay for transposase-accessible chromatin with high-throughput sequencing and RNA-seq analyses indicated that transcriptional activity of the genes strongly correlates with chromatin accessibility. Chromatin transcription-activated gene expression regulation is dominant during high-temperature stress in *B. tabaci*, mainly through the transcriptional repression of genes related to low-temperature stress resistance. Furthermore, *B. tabaci* resists low-temperature stress by regulating enzyme activities and withstands high-temperature stress by regulating metabolism and synthesis of organic substances, both achieved by altering chromatin accessibility. In summary, this study provides a theoretical basis for exploring changes in gene expression and chromatin accessibility under different temperature stresses, offering a new approach to unravelling regulatory mechanisms underlying the onset of molecular regulation in response to various temperature stress conditions.

## 1. Introduction

The invasive species *Bemisia tabaci* is characterized by its rapid reproduction speed, substantial egg production, considerable generational overlapping, and ability to spread various viruses. It has spread worldwide, causing outbreaks in many countries, including India, the United States, and China. Additionally, it has become an important pest that seriously damages vegetables and garden plants. Global warming is a universal threat to all species and ecosystems [1]. Insects are ectotherms, whose behavior and physiology are heavily dependent on environmental conditions [2]. Ambient temperature affects physiological activities of ectothermic animals to a greater or lesser extent. Therefore, temperature tolerance in insects is critical for the existence and persistence of their populations.

Insect’s geographical distribution and dispersion, especially invasive insects, are largely dependent on their temperature stress tolerance. Species such as *Liriomyza* spp., *Hyphantria cunea*, and *B. tabaci* can adapt to new conditions, allowing them to spread from their origins and establish populations in new countries with different abiotic conditions, such as temperature [3,4,5]. Therefore, these organisms usually have strong invasiveness and wider distribution ranges, resulting in stronger resistance to varied temperatures, rendering them difficult to control. Exposure of widely dispersed species to different ambient temperatures often results in varied phenotypic selection and intraspecific variation. Intraspecific variations arise via genetic mechanisms or phenotypic plasticity [6]. Previous studies show these variations occur frequently [7,8,9]. With global warming, thermal tolerance is an important factor influencing intraspecific variation. However, the molecular regulatory mechanism has not yet been determined.

Epigenetics is the level of gene expression changes without changes to gene sequence, and it has gained interest in research. The scope for epigenetic research is extensive, including DNA methylation, histone modification, variable RNA splicing, miRNA regulation, transcription regulation, chromatin accessibility, etc. Recently, epigenetics was proposed to have a significant effect on temperature tolerance [10,11,12]. Chromatin accessibility, also known as chromatin openness, reflects transcriptional activity of chromatin, and it is a crucial aspect for studying epigenetic regulation. Recently, many studies on the role epigenetics plays in temperature stress have emerged, and its impact on the temperature adaptation process in organisms is being gradually unveiled [10,11,12]. For instance, a short-term epigenetic mechanism is an important driving factor in *Aedes albopictus* to increase cold tolerance and spread to high altitudes and latitudes [13]. Knockdown of DNA methyltransferase and chromatin remodeling factor ISWI expression significantly affects temperature tolerance and decreased high- and low-temperature tolerance of *B. tabaci* [14,15]. However, current research mainly focuses on single key factors in epigenetic regulation. A systematic study on the epigenetic regulation network is necessary to comprehensively dissect the mechanisms in organisms that allow them to rapidly adapt to environmental temperature changes.

This study explores how chromatin accessibility influences temperature tolerance to yield various phenotypes under environmental temperature stress in ectothermic organisms, such as the whitefly, using RNA-seq to reveal differences in gene expression levels among three different temperatures. This study also establishes different global chromatin accessibility profiles using an assay for transposase-accessible chromatin with high-throughput sequencing (ATAC-seq) [16]. A combined analysis using RNA-seq and ATAC-seq data was performed to investigate how chromatin accessibility affects transcriptome changes. Thus, key genes in temperature adaptation and regulatory pathways could be identified. These results provide insights into the molecular mechanisms underlying temperature stress responses.

## 2. Materials and Methods

### 2.1. Insect Rearing

*B. tabaci* cryptic species Middle East-Asia Minor 1 (MEAM1) was reared on cotton plants in greenhouse conditions. The insects were maintained within cages in an insectary at 24–27 °C with 50–60% relative humidity and a 14:10 h light–dark cycle.

### 2.2. Material Preparation

We provided cotton plants in the cage for *B. tabaci* adults to lay eggs on for three days, and we moved these cotton plants from each day into an incubator set at 21 °C, 26 °C, and 31 °C, respectively. These temperatures were the lowest, optimal, and highest for which the whitefly can complete its life cycle and maintain long-term development of the population. We cultivated the eggs until adult emergence within 72 h, and the adult was then collected (both male and female). Three replicates were set for each treatment, named B21-1, B21-2, B21-3; B26-1, B26-2, B26-3; B31-1, B31-2, and B31-3.

### 2.3. RNA-Seq Library Preparation, Sequencing, and Analysis

Seq health Technology Co., Ltd. (Wuhan, China) conducted the UID RNA-seq experiment, i.e., high-throughput sequencing. Total RNA was extracted from the above treatments using TRIzol Reagent (Invitrogen, Carlsbad, CA, USA, Cat. No. 15596026) according to the protocol in [17]. RNA quality and integrity were detected using a Nanodrop TM One Spectrophotometer (Thermo Fisher Scientific Inc., IMPLEN, Munich, Germany) and 1.5% agarose gel electrophoresis. Qualified RNA samples were then quantified by using Qubit3.0 with the Qubit TM RNA Broad Range Assay Kit (Life Technologies, Carlsbad, CA, USA). Thereafter, 2 μg of total RNA was used for the stranded RNA sequencing library preparation using a KC-Digital TM Stranded mRNA Library Prep Kit for Illumina^®^ (Catalog No. DR08502, Wuhan Seq health Co., Ltd., Wuhan, China). This eliminates the duplication bias in PCR and sequencing steps by using a unique molecular identifier (UMI, Tokyo, Japan) of eight random bases to label pre-amplified cDNA molecules, according to the manufacturer’s instructions. The library products (200–500 bps) were enriched, quantified, and sequenced on the Novaseq 6000 sequencer (Illumina, San Diego, CA, USA) PE150 model.

Trimmomatic (version 0.36) was used to filter the raw data [18]. Clean reads were further treated using FastUniq (version 1.1) to remove duplication bias [19]. The deduplicated sequences were used for standard RNA-seq analysis, and were mapped to the *B. tabaci* MEAM1 reference genome [20] using STAR software (version 2.5.3a) [21]. Reads mapped to the exon regions were counted by using the feature Counts (Subread-1.5.1; Bioconductor), and the reads per kilobase million (RPKM) were then calculated [22]. The RPKM method can eliminate the influence of differences in gene length and sequencing quantity when calculating gene expression, and the calculated gene expression quantity can be directly used to compare gene expression differences between different products. If there are multiple transcripts of a gene, the longest transcript of the gene is used to calculate its sequencing coverage and expression level. The significant differences in the gene expression level between groups were identified using the R package Edge (version 3.12.1) [23]. The parameters for establishing significance were *p*-value < 0.05 and fold change >2. KOBAS software (version: 2.1.1) was used for Gene ontology (GO) and Kyoto Encyclopaedia of Genes and Genomes (KEGG) enrichment analysis of DEGs [24].

### 2.4. ATAC-Seq Library Preparation, Sequencing, and Analysis

The ATAC assay, high-throughput sequencing was conducted by Seq Health Technology (following Section 2.3). Three hundred *B. tabaci* MEAM1 adults in each replicate were frozen in liquid nitrogen and ground using a Tissuelyser (Tissuelyser-24, Shanghai Jingxin, Shanghai, China). The ground powder was treated with a cell lysis buffer, and nuclei were collected via centrifugation for 5 min at 2000× *g*. Transposition and high-throughput DNA sequencing library preparation were conducted using the True Prep DNA Library Prep Kit V2 for Illumina (Catalog No. TD501, Vazyme, Nanjing, China). Library products were enriched, quantified, and sequenced using the Novaseq 6000 sequencer (Illumina, San Diego, CA, USA) PE150 model.

Raw sequencing data were filtered using Trimmomatic (version 0.36), low-quality reads were discarded, and adaptor sequences were trimmed. Fast Uniq (version 1.1) was used to eliminate duplication of clean reads. The reads were then mapped to the *B. tabaci* MEAM1 reference genome using bowtie2 software (version 2.2.6) with default parameters [25]. Reads mapped to the mitochondrial genome were filtered with in-house scripts (https://github.com/samtools/samtools, accessed on 12 December 2019). Read distribution analysis was completed on RseQC (version 2.6) [26]. The Collect Insert Size Metrics tool from Picard software (version 2.8.2) was used to count the insert length. Peak calling was performed on MACS2 software (Version 2.1.1), while peak annotation and distribution analyses were performed on bedtools (Version 2.25.0) [27,28]. Three replicates were used to identify credible peaks. The peaks were identified with bedtools (Version 2.25.0) using the Fisher test [29]. The method and parameters for enrichment analysis for annotated genes were the same as in Section 2.3.

### 2.5. Real-Time Quantitative PCR (RT-qPCR) Analysis

RT-qPCR was used to validate the sequencing data. The qPCR reactions were performed on an ABI 7500 real-time PCR system (Applied Biosystems, Waltham, MA, USA). The analysis preparation and program parameters can be found in our previous publications [15]. All batches included controls without the cDNA template. The mRNA relative expression level was calculated using a mathematical model (2^–ΔΔCT^) [30]. The RT-qPCR primers used are listed in Supplementary Table S1.

## 3. Results

### 3.1. Genome-Wide Gene Expression Changes under Different Temperature Stresses

**Figure 1:**
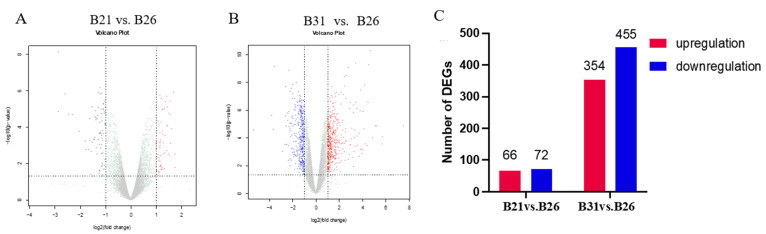
RNA−seq revealing transcriptome dynamics under different stress temperatures. (**A**,**B**) Volcano plots showing significantly up− and downregulated gene expressions from B21 vs. B26 and B31 vs. B26. The gray dots represent genes that have not undergone differential expression, the blue dots represent genes that have undergone differential downregulation, and the red dots represent genes that have undergone differential upregulation. (**C**) Number of differentially expressed genes (DEGs) for each pairwise comparison.

The mRNA pools from each of the stress temperatures were subjected to RNA-seq to detect the gene expression landscape under different temperature stresses. More than 37.1 million clean reads were generated by each library (Supplementary Table S2); B21, B26, and B31 deduplication unique ratios varied from 72.15% to 75.13%, 73.59% to 76.42%, and 72.82% to 76.24%, respectively, indicating good reproducibility. Over 90% of the clean reads were uniquely mapped to the *B. tabaci* reference genome. Additionally, nine RNA-seq libraries could be grouped into three distinct clusters by using hierarchical clustering analysis (Supplementary Table S3 and Supplementary Figure S1). B21 and B26 showed similar expression patterns, likely because 21 °C falls within the range of suitable temperatures for the whitefly. Despite this, the above findings indicated good repeatability of our RNA-seq data. The gene expression landscape under different temperature stress conditions was revealed via quantitative RPKM-based analysis (Figure 1A,B). Candidate genes (significantly over- and underexpressed) were screened based on the same threshold for each pairwise comparison. In detail, 66 and 354 overexpressed genes and 72 and 455 underexpressed genes were identified between B21 and B26 and between B31 and B26, respectively (Figure 1C). The target gene responses to different temperature stresses were found based on these transcriptome data.

### 3.2. Enrichment Analysis of DEGs under Different Temperature Stresses

**Figure 2:**
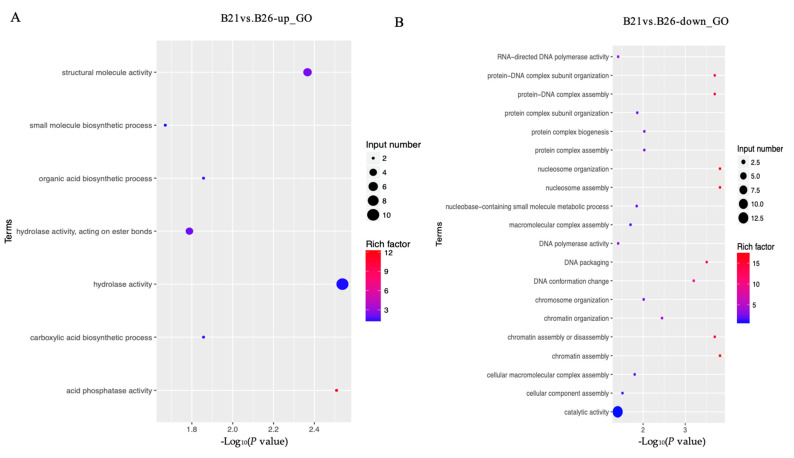

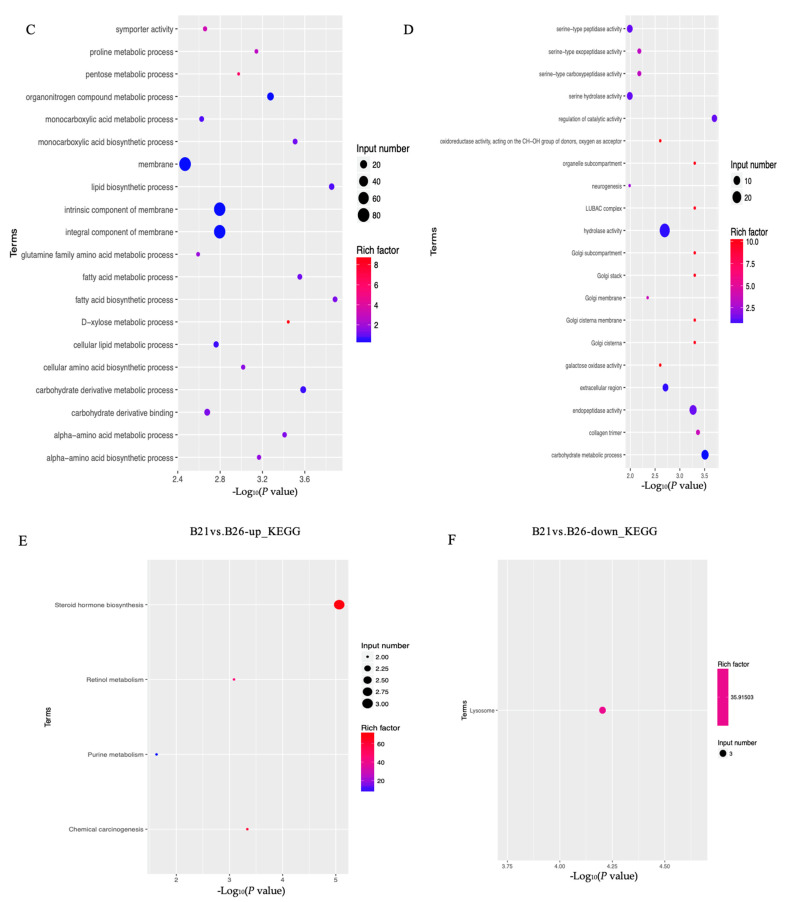

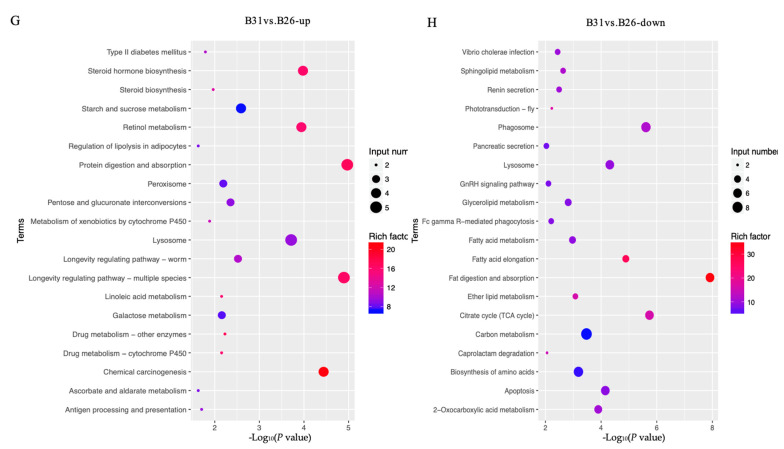
Gene Ontology (GO) and Kyoto Encyclopaedia of Genes and Genomes (KEGG) analyses for differentially expressed genes (DEGs) between different stress temperatures. (**A**–**D**) Top GO categories of biological processes enriched by DEGs from B21 vs. B26 and B31 vs. B26, respectively. (**E**–**H**) Top KEGG pathways enriched by DEGs from B21 vs. B26 and B31 vs. B26.

The top GO categories for biological processes (BPs) enriched by DEGs under different temperature stresses are displayed in Figure 2. The majority of the DEGs identified from the comparison between B21 and B26 were enriched in relation to structural molecule, hydrolase, and catalytic activities. Several significantly enriched terms between B26 and B31 were related to regulation of catalytic activity, hydrolase activity, extracellular region, endopeptidase activity, and carbohydrate metabolic processes. Most DEGs between B21 and B26 were enriched in the lysosome and the steroid hormone biosynthesis KEGG pathways. Those between B26 and B31 were enriched in steroid hormone biosynthesis, retinol metabolism, protein digestion, absorption, lysosome, the longevity-regulating pathway (multiple species), chemical carcinogenesis, phagosome, fatty acid elongation, fat digestion and absorption, and the citrate cycle (TCA cycle).

### 3.3. Establishing the Open Chromatin Landscape

**Figure 3:**
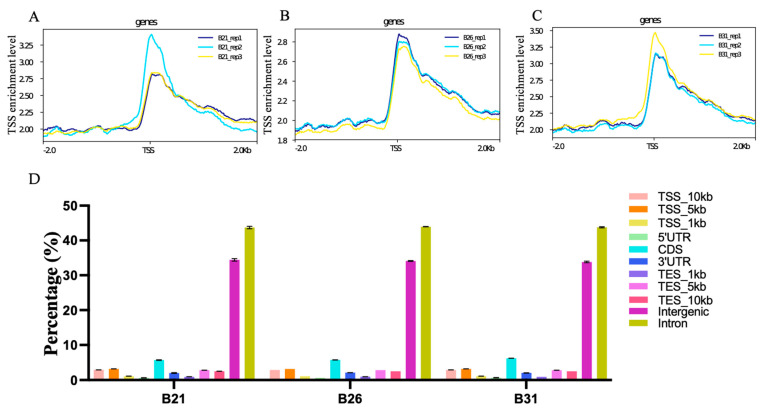
Quality estimation, peak calling, and genomic distribution of the ATAC-seq reads under different stress temperatures. (**A**–**C**) Distribution plots of sequencing reads from a representative ATAC-seq library across all genes. (**D**) Number and genomic distribution of peaks identified by ATAC-seq in each sample.

**Figure 4:**
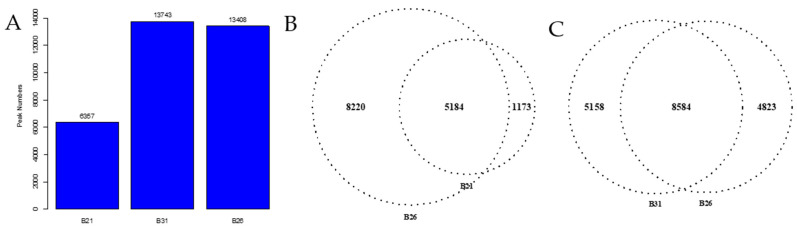

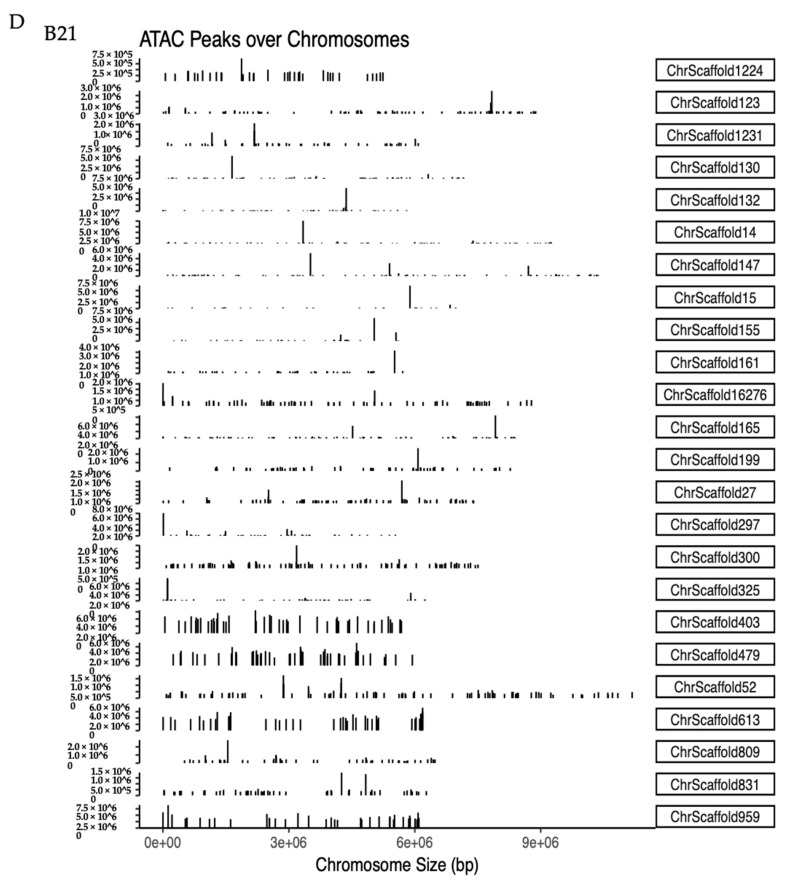

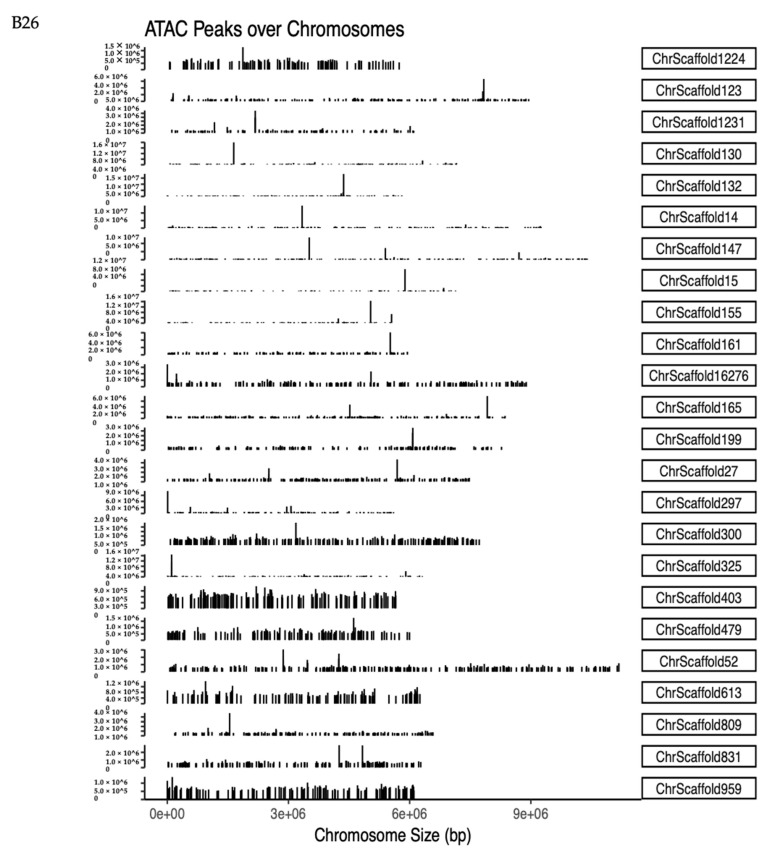

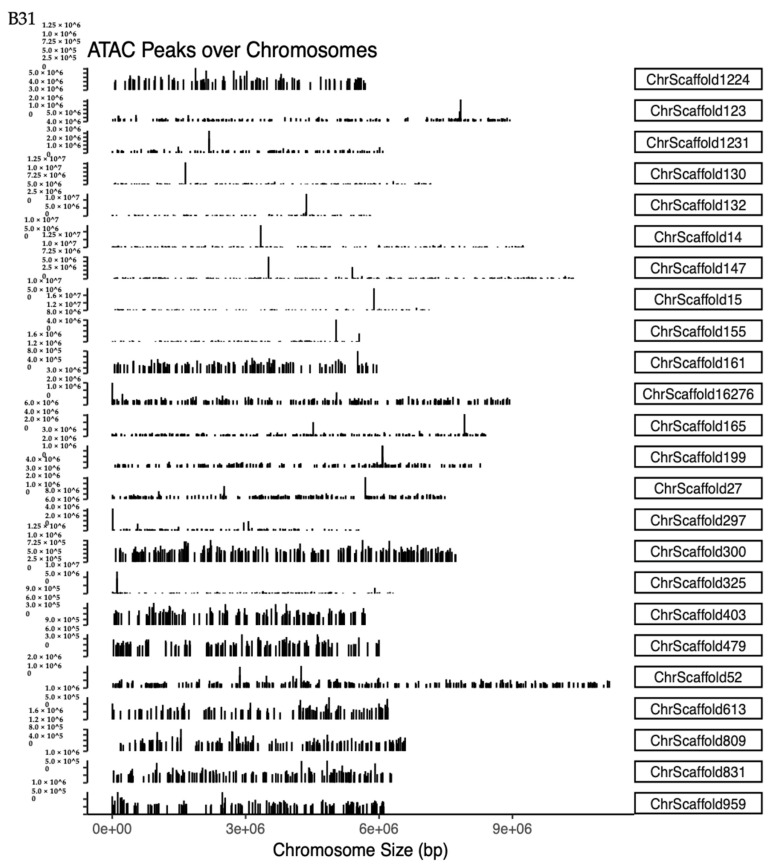

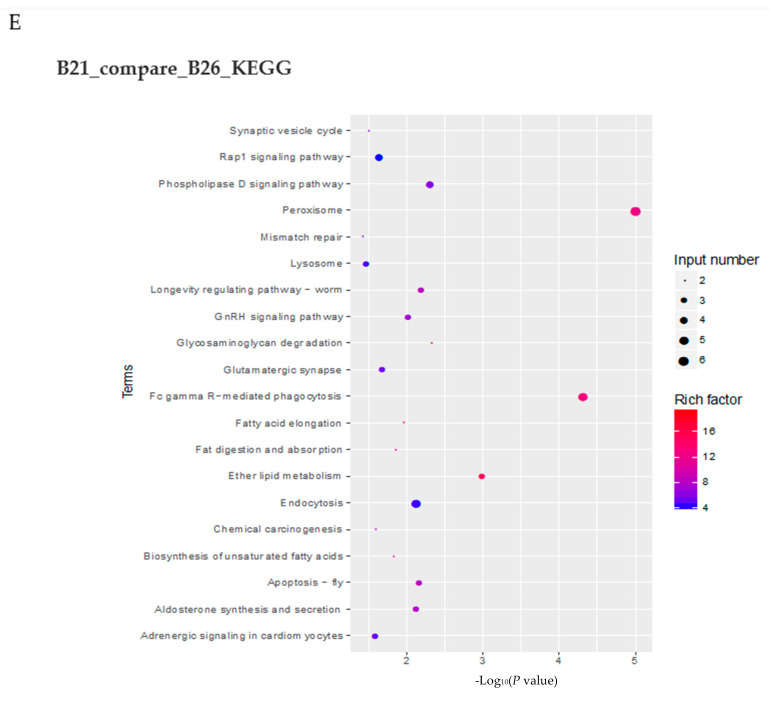

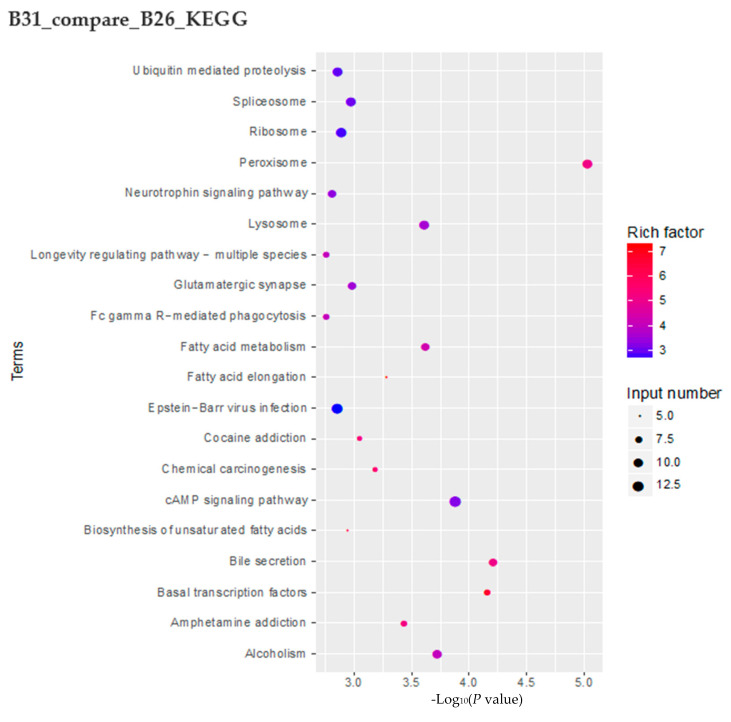

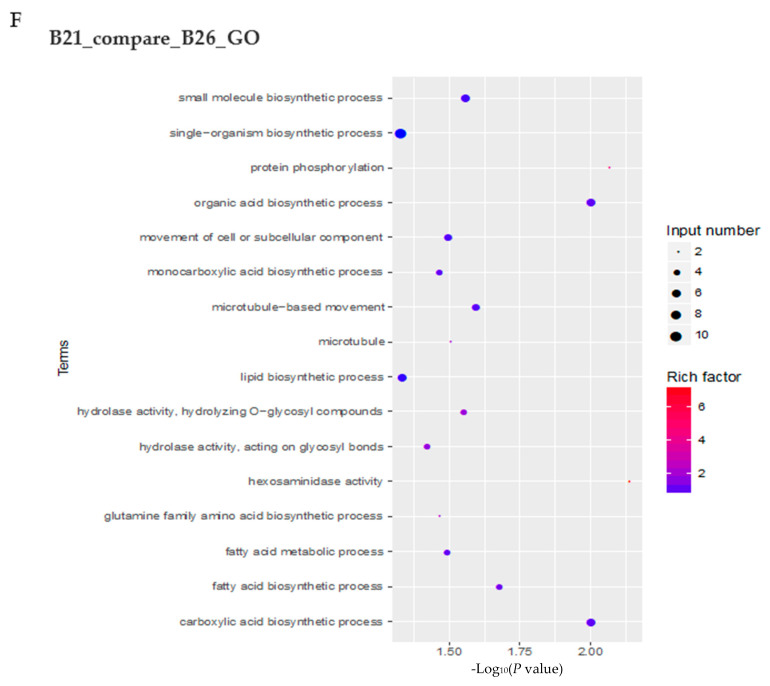

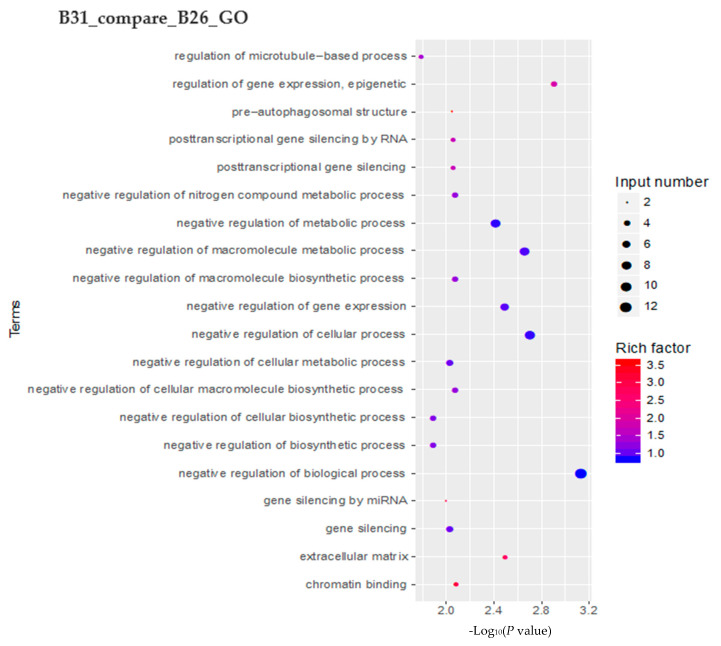
Changes in accessible chromatin and enrichment of genes by differential peaks under different stress temperatures. (**A**) Number of peaks in B21, B26, and B31. (**B**,**C**) Venn diagrams showing the number of differential peaks in B21 vs. B26 and B31 vs. B26. (**D**) ATAC peaks over chromosomes in B21, B26, and B31. The abscissa represents the length of the chromosome, the right side represents the chromosome number, and the left ordinate represents the depth of coverage. (**E**) Top Kyoto Encyclopaedia of Genes and Genomes (KEGG) pathways enriched by genes near differential peaks from B21 vs. B26 and B31 vs. B26. (**F**) Top Gene Ontology (GO) categories of biological processes enriched by genes near differential peaks from B21 vs. B26 and B31 vs. B26.

The pooled suspensions from each of the three representative stress temperatures were subjected to ATAC-seq to define the chromatin accessibility landscape. Over 30.3 million clean reads were obtained in each library, and the GC content ranged from 42% to 46%. Approximately 70% of the clean reads from each library were uniquely mapped to the *B. tabaci* reference genome. These results are detailed in Supplementary Table S3. The ATAC-seq signal was stronger at the transcription start site (TSS) (Figure 3A–C), indicating that most ATAC-seq reads were distributed around the TSS. Peak annotation suggested that over 40% and 30% of the peaks from each sample were located in introns and intergenic regions, respectively; the percentages of peaks from each sample located in TSS_1kb–10kb, 5′UTR, CDS, 3′UTR, and TES_1kb–10kb are shown in Figure 3D. As shown in Figure 4A, 6357, 13408, and 13743 peaks were identified in B21, B26, and B31, respectively. Most peaks spanned 350–400 bps. It is worth noting that peaks observed by three biological replicates are defined as differential peaks (DPs) for each temperature treatment, while peaks detected in only one biological replicate are interfering peaks. Therefore, the above results implied high quality and reproducibility of the sequence data in this study.

### 3.4. Differences in Open Chromatin under Different Temperature Stresses

Identified peaks under different temperature stress conditions were compared using a Python script to explore chromatin accessibility changes. The number of peaks detected at 21 °C was approximately half that of the other two treatment groups (B26 and B31) (Figure 4A), suggesting that more chromatin sites are turned off during low-temperature stress. About 18% unique peaks were detected in the 21 °C treatment groups and 36% unique peaks in the 31 °C treatment groups, as shown in Figure 4B,C. This suggests that although chromatin accessibility is altered, it is not identical under high- and low-temperature stress. Additionally, chromatin openness was significantly reduced under low-temperature stress but significantly increased under high-temperature stress (Figure 4D). These results further confirm that different chromatin regulation patterns occur at high and low temperatures. This demonstrates significant differences in chromatin accessibility under different temperature stresses, similar to changes in the transcriptome, sustaining the hypothesis that differential chromatin accessibility may alter the gene expression profiles in response to temperature.

### 3.5. Enrichment Analysis of Genes with Differential Peaks (DPs) under Different Temperature Stresses

KEGG enrichment analysis was performed on genes with DPs around their CDS, 3′ UTR, and 5′ UTR regions. Genes with B21-specific peaks were mainly enriched in peroxisome, Fc gamma R-mediated phagocytosis, ether lipid metabolism, glycosaminoglycan degradation, and phospholipase D signaling pathway (Figure 4E). Genes with B31-specific peaks were mainly enriched in peroxisome, bile secretion, basal transcription factors, cAMP signaling pathway, alcoholism, and fatty acid metabolism (Figure 4E).

The GO categories for biological processes enriched by genes with B21- and B31-specific peaks are displayed in Figure 4E. Genes with B21-specific peaks were mainly enriched in the single-organism, organic acid, carboxylic acid, small-molecule, and lipid biosynthetic processes. In comparison, genes associated with B31-specific peaks were mainly enriched in the biological processes of negative regulation, the cell surface receptor signaling pathway, negative regulation of cellular processes, negative regulation of macromolecule metabolic processes, and negative regulation of metabolic processes. These results imply that the whitefly mainly relies on synthesizing acids, small molecules, and lipids to withstand the low-temperature adversity. The signal transduction and material metabolism regulation is the main measure by which the whitefly responds to high-temperature stress.

### 3.6. Association between Chromatin Accessibility and Transcriptome under Different Temperature Stresses

**Figure 5:**
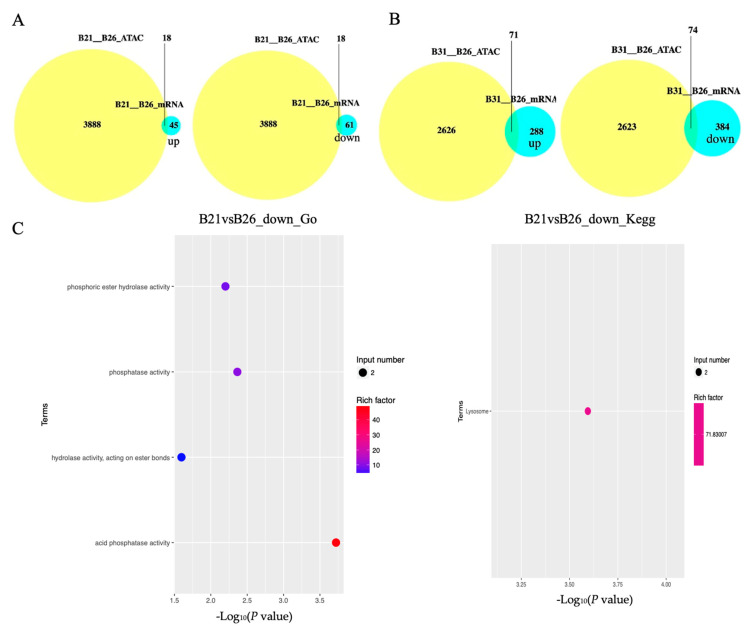

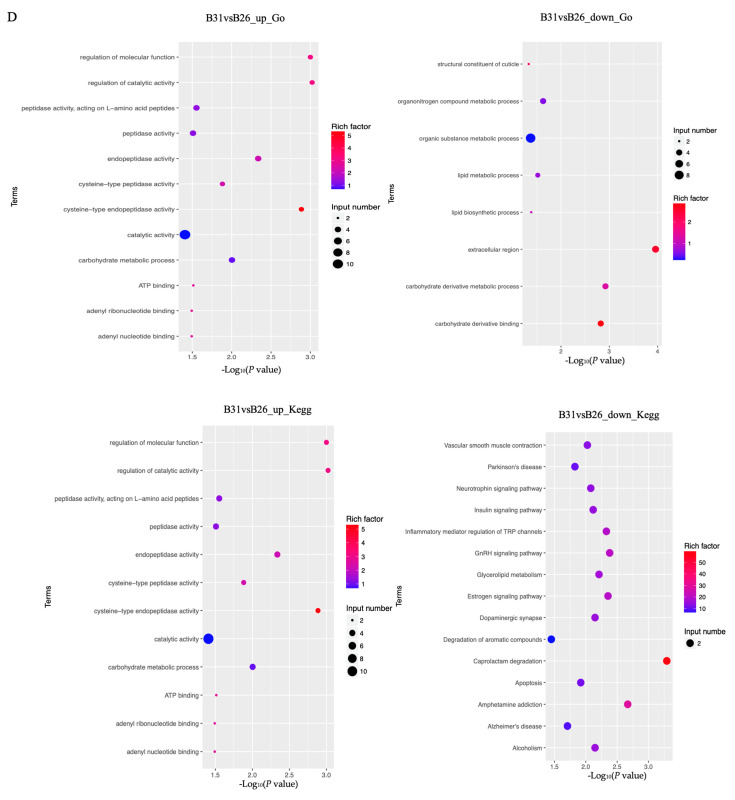
Correlation analysis between differentially expressed genes (DEGs) and differential peaks. (**A**,**B**) Venn diagrams showing the number of DEGs with differential peaks in B21 vs. B26 and B31 vs. B26. The absolute value of log FC > 1 and *p*-value < 0.05 were used as the standard to identify DEGs. (**C**,**D**) Top Gene Ontology (GO) categories of biological processes and Kyoto Encyclopaedia of Genes and Genomes (KEGG) pathways enriched by DEGs with differential peaks from B21 vs. B26 and B31 vs. B26.

ATAC-seq and RNA-seq data were integrated and analyzed to determine whether the open chromatin regions’ response to temperature stress was related to changes in gene expression patterns. Genes with DPs of ATAC-seq and DEGs of mRNA-seq were used as intersections to obtain genes affected by chromatin accessibility. Figure 5A,B show that approximately 40% of all upregulated genes and 30% of all downregulated genes in B21 were regulated by chromatin accessibility, while this was approximately 25% of all upregulated genes and 19% of all downregulated genes in B31. These results indicate that no more than 40% of the genes have altered transcriptional expression levels due to chromatin accessibility. Firstly, the results show that differentially accessible sites do not substantially affect RNA levels. Secondly, mechanisms other than chromatin remodeling might regulate gene expression.

A KEGG enrichment analysis was performed on these genes. The results are displayed in Figure 5C,D. When comparing between B21 and B26, we found that the lysosome was the only enriched pathway by downregulated genes around DPs. The five most significantly enriched pathways by downregulated genes around DPs, when comparing B31 and B26, were caprolactam degradation, amphetamine addiction, GnRH signaling pathway, estrogen signaling pathway and inflammatory mediator regulation of TRP channels, whereas those by overexpressed genes around DPs were the lysosome, longevity-regulating pathway (multiple species), antigen processing and presentation, fatty acid degradation, and fatty acid metabolism.

The GO categories for molecular functions enriched by the downregulated genes around DPs between B21 and B26 were acid phosphatase, phosphatase, phosphoric ester hydrolase, and hydrolase activity (Figure 5C,D). The GO categories for biological processes by the overexpressed genes around DPs between B31 and B26 were the organic substance, carbohydrate derivative, organonitrogen compound, lipid metabolic processes, and lipid biosynthetic process. The GO categories for biological processes by the downregulated genes around DPs between B31 and B26 were the carbohydrate metabolic process, regulation of catalytic activity, and regulation of molecular function, while those of molecular function were catalytic and endopeptidase activity. Therefore, we concluded that GO categories were enriched by nearby DEGs of DPs under low-temperature stress, with the activity of several enzymes, and by DEGs of DPs under high-temperature stress, with two aspects including organic substance metabolic processes and enzymatic activities. This shows that whiteflies mainly rely on regulating enzyme activity in response to low-temperature stress, and regulating metabolism of organic substances (including lipids) to resist high-temperature stress. Combined with the enrichment results of KEGG, it can be concluded that lipid metabolism and synthesis play a vital role under high-temperature stress.

### 3.7. Expression Patterns of Key Candidate Genes under Temperature Stress

**Figure 6:**
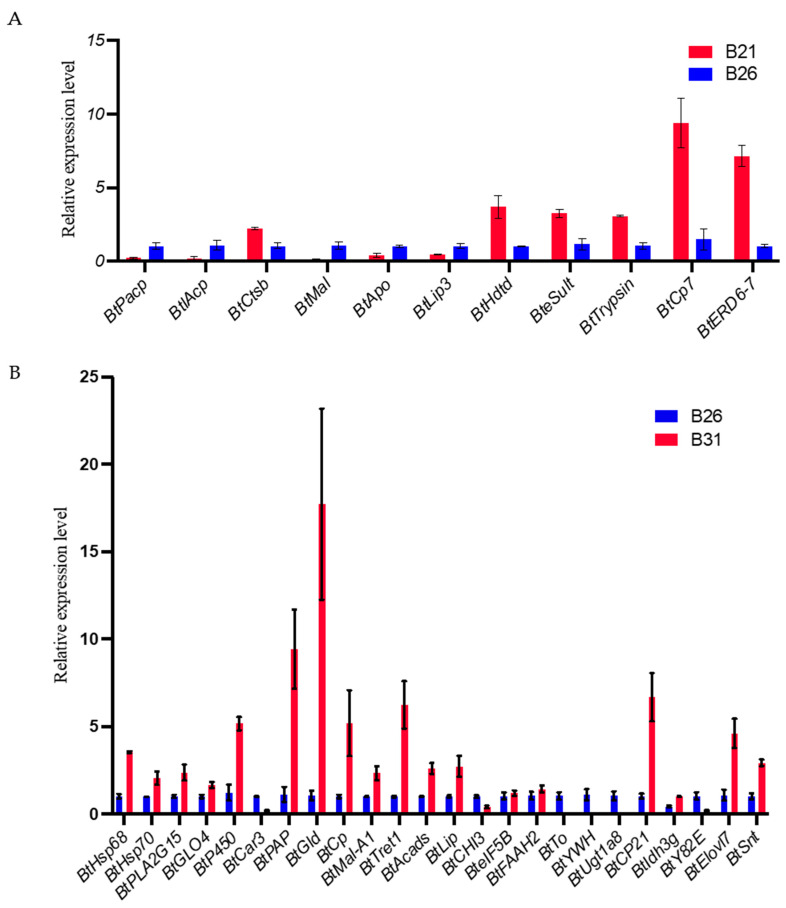
Real-time quantitative PCR (RT-qPCR) validation for expression of the main differentially expressed genes (DEGs) with differential peaks under different stress temperatures. The RT-qPCR results are expressed as the mean ± SEM of the three groups. (**A**) RT-qPCR validation of expression of the main DEGs with differential peaks between B21 and B26. (**B**) RT-qPCR validation of expression of the main DEGs with differential peaks between B31 and B26.

**Figure 7:**
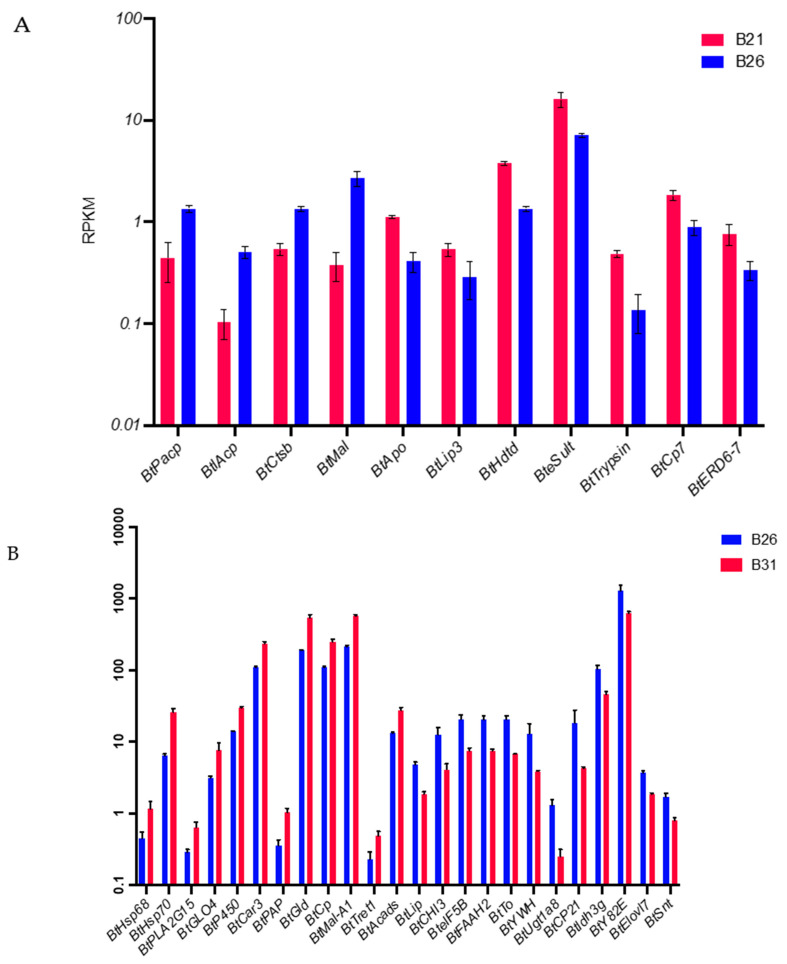
RPKM values of the main differentially expressed genes (DEGs) with differential peaks under different stress temperatures. The RPKM values were converted to log10. (**A**) RPKM values of the main DEGs with differential peaks between B21and B26. (**B**) RPKM values of the main DEGs with differential peaks between B31and B26.

Based on the data from Section 2.4, 11 and 26 genes from the comparisons between B21 and B26 and between B31 and B26, respectively, were obtained for qPCR validation after excluding genes for uncharacterized proteins. The full names and putative functions of these genes are shown in Table 1. As shown in Figure 6A,B, the expression level of almost 90% of the candidate genes detected by qPCR shows the same change that was observed using RNA-seq in Figure 7A,B, demonstrating the reliability of the sequence method selected in this study.

## 4. Discussion

RNA-seq and ATAC-seq data were integrated and analyzed to unravel the regulatory network response to temperature stress. Our results indicate that transcriptional alterations of the target genes are closely related to chromatin accessibility of functional genomic regions. Furthermore, chromatin openness was significantly reduced at low temperatures and significantly increased at high temperatures. Additionally, our results demonstrate that the chromatin transcription-activated gene expression regulation is dominant under high-temperature stress in *B. tabaci*, mainly through repression of the gene expression to resist low-temperature stress. Notably, DEGs correlated with chromatin accessibility from high-temperature stress almost completely differed from DEGs correlated with low-temperature stress. DEGs from low-temperature stress contained several enzymes (phosphatase, cathepsin, etc.). Thus, a string of key transcriptional regulation modes regulated by chromatin accessibility were further demonstrated in this study in response to temperature stress.

Alternate adaptation mechanisms for genetic alteration must exist in organisms, as many environmental stressors can only persist for a limited time. Reversible epigenetic modifications that regulate gene expression without altering DNA sequence have emerged as an attractive mechanism for transcriptional regulation. Additionally, recent studies suggest that it has been implicated in regulating stress-related gene expression [11]. Such organisms, able to temporarily and rapidly alter gene expression, have an evolutionary advantage, which is crucial for their survival. Temperature adaptation often involves numerous transcriptional regulatory processes [31,32], which are partly affected by dynamic chromatin alterations. Therefore, changes in chromatin accessibility are an important molecular response to stress.

DEGs from high-temperature stress not only included heat shock protein 70 and cytochrome P450 as expected [33,34,35] but also several enzymes involved in lipid synthesis and metabolism, such as carbonic anhydrase 3, facilitated trehalose transporter, chitinase 3, and noradrenaline transporter. These key genes are involved in processes such as sugar conversion and transportation, as well as apoptosis. Carbonic anhydrases have been cloned in several species, such as *Drosophila melanogaster* and *Caenorhabditis elegans* [36,37]. This enzyme is localized in mitochondria and likely involved in metabolic processes that participate in gluconeogenesis, lipogenesis, and ureagenesis for insects [38]. Tret1 is a trehalose-specific facilitated transporter, and its expression is stress-induced, thus indicating that *Tret1* participates in trehalose transport in cells. Our findings suggest that *B. tabaci* resists low-temperature stress by modulating activities of several enzymes, whereas it withstands high-temperature stress by modulating metabolism and synthesis of organic substances. Additionally, these findings suggest that sugar conversion and transportation have an important effect on insects’ temperature adaptation as well as the two biological processes in which heat shock proteins and cytochrome P450 are involved. Moreover, all three biological processes are regulated by chromatin accessibility.

This study demonstrates that regulation of genes by chromatin accessibility, an epigenetic regulatory mechanism, has a vital effect on the temperature tolerance in *B. tabaci*. This study demonstrates the accessible chromatin changes in *B. tabaci* under temperature stress for the first time, to the best of our knowledge. Gene expression can be activated or repressed by mobilizing chromatin openness of unique genomic regions that perform specific functions. The present study reveals the deep-level temperature tolerance mechanism of *B tabaci*, thus providing a new understanding of its temperature adaptation and rapid expansion mechanism, and providing a theoretical basis for exploring new technologies for its innovative intervention and control.

## 5. Conclusions

This study used RNA-seq and ATAC-seq to reveal differences in global gene expression patterns and chromatin accessibility landscapes under temperature stress in *B. tabaci*, and it indicated the relationship between dynamic transcriptomic changes and chromatin accessibility, as well as identified key pathways and genes involved in temperature adaptation. However, the precise functions of the elements need to be further studied using CRISPR/Cas9 and RNAi methods.

## Figures and Tables

**Table 1:** Information for target genes.

Abbreviated Name	Full Gene Name	Putative Functions	Change in Expression Level
*BtpAcp*	prostatic acid phosphatase	histidine phosphatase activity	Downregulated at 21 °C
*BtlAcp*	lysosomal acid phosphatase	histidine acid phosphatase and phytase activity	
*BtCtsb*	cathepsin B	proteolysis involved in cellular protein catabolic process	
*BtMal*	maltase	maltose alpha-glucosidase activity	
*BtApo*	aromatic peroxygenase	peroxidase activity	Upregulated at 21 °C
*BtLip3*	lipase 3	triglyceride lipase activity; involved in lipid catabolic process	
*BtHdtd*	hydroxyacid-oxoacid transhydrogenase	involved in glutamate catabolic process via 2-oxoglutarate	
*BteSult*	estrogen sulfotransferase	sulfotransferase activity	
*Bttrypsin-1*	trypsin-1	trypsin-like serine protease activity	
*BtCp7*	cuticle protein 7	insect cuticle protein	
*BtERD6-7*	sugar transporter ERD6-like 7	glucose transporter-like family of the Major Facilitator superfamily of membrane transport proteins	
*BtHsp68*	heat shock protein 68	ATP hydrolysis activity	Upregulated at 31 °C
*BtHsp70*	heat shock protein 70	Involved in heat-shock-mediated polytene chromosome puffing and response to hypoxia	
*BtPLA2G15*	group XV phospholipase A2	alpha/beta hydrolase activity	
*BtGLO4*	hydroxyacylglutathione hydrolase	hydroxyacylglutathione hydrolase activity	Upregulated at 31 °C
*BtP4504C1*	Cytochrome P450 4C1	catalyze a variety of oxidative reactions of a large number of structurally different endogenous and exogenous compounds in organisms	
*BtCar3*	carbonic anhydrase 3	regulate pH homeostasis	
*BtPAP*	prostatic acid phosphatase-like	histidine phosphatase activity	
*BtGld*	glucose dehydrogenase	oxidoreductase activity	
*BtCp*	cysteine proteinase		
*BtMal-A1*	maltase A1	predicted to be involved in carbohydrate metabolic process	
*BtTret1*	trehalose transporter 1	transport of trehalose synthesized in the fat body and the incorporation of trehalose into other tissues that require a carbon source	
*BtAcads*	short-chain specific acyl-CoA dehydrogenase	involved in fatty acid beta-oxidation using acyl-CoA dehydrogenase	
*BtLip*	gastric triacylglycerol lipase	triglyceride lipase activity; involved in lipid catabolic process	Downregulated at 31 °C
*BtCHI3*	chitinase 3	chitinases catalyze the hydrolysis of chitin	
*BteIF5B*	eukaryotic translation initiation factor 5B	predicted to be involved in translational initiation	Downregulated at 31 °C
*BtFAAH2*	fatty-acid amide hydrolase 2	Asp-tRNAAsn/Glu-tRNAGln amidotransferase A subunit or related amidase (translation, ribosomal structure, and biogenesis)	
*BtTo*	takeout protein	behavioral response to starvation	
*BtYWH*	tyrosine 3-monooxygenase	enables transcription factor binding	
*BtUgt1a8*	UDP-glucuronosyltransferase 1-8	an enzyme of the glucuronidation pathway that transforms small lipophilic molecules, such as steroids, bilirubin, hormones, and drugs, into water-soluble, excretable metabolites	
*BtCP21*	cuticle protein 21	insect cuticle protein	
*BtIdh3g*	isocitrate dehydrogenase [NAD] subunit gamma	carboxydipeptidyl activity	
*BtY82E*	ATP synthase lipid-binding protein	enables ATP binding, involved in carbohydrate metabolic process	
*BtElovl7*	elongation of very long chain fatty acids protein 7	enables fatty acid elongase activity; involved in fatty acid elongation and polyunsaturated fatty acid and very-long-chain fatty acid biosynthetic process	
*BtSnt*	sodium-dependent noradrenaline transporter	involved in neurotransmitter transport	

## Data Availability

The RNA-Seq and ATAC-Seq of *Bemisia tabaci* data (CNP0004906) that support the findings of this study are openly available in CNSA of CNGBdb at https://db.cngb.org/cnsa/, accessed on 17 October 2023.

## Supplementary Materials

The following supporting information can be downloaded at: https://www.mdpi.com/article/10.3390/genes14101978/s1, Figure S1: The Cluster heatmap formed by differentially expressed genes; Table S1: Primer information for qRT-PCR validation of expression of the main differentially expressed genes (DEGs) with differential peaks around their CDS, 3′ UTR, or 5′ UTR regions under different temperature stress; Table S2: UID deduplication basic data statistics for RNA-seq; Table S3: Assay for Transposase-Accessible Chromatin with high-throughput sequencing (ATAC-seq) data quality control statistics.
