# Supplementary material for: Characteristics of the Accessible Chromatin Landscape and Transcriptome under Different Temperature Stresses in Bemisia tabaci
Source: Genes (Basel). 2023 Oct 23;14(10):1978. doi: 10.3390/genes14101978 (PMC10606294; doi:10.3390/genes14101978)
Supplement: Supplementary file 1 [file genes-14-01978-s001.zip › genes-2643111-supplementary.docx]

**Supplementary Table S1.** Primer information for qRT-PCR validation of expression of the main differentially expressed genes (DEGs) with differential peaks around their CDS, 3' UTR, or 5' UTR regions under different temperature stress.

| Gene name | Primer name | Sequence information（5'-3'） |
| --- | --- | --- |
| *BtpAcp* | pAcp-F | CTGTGAACTACTCGCCG |
|  | pAcp-R | CCATTGCTTCCTCTCTTG |
| *BtlAcp* | lAcp-F | GTTCCTCTCGGCTAAAT |
|  | lAcp-R | AACCAACGATAACCACTC |
| *BtCtsb* | Ctsb-F | GAGAAAACAATGCCCGA |
|  | Ctsb-R | CGCAGAAGTAGCAACAAGA |
| *BtMal* | Mal-F | GGGACAACAAAACAAGA |
|  | Mal-R | GACCAACCACGAATAAT |
| *BtApo* | Apo-F | TAGCCACCGTTCCTCAGTT |
|  | Apo-R | CAGCCATAGCCTCCCAAT |
| *BtLip3* | Lip3-F | TTTGGGATTTCAGTTGG |
|  | Lip3-R | GTATTCGGGTAGCGTTG |
| *BtHdtd* | Hdtd-F | GAAAAGGAAAACCAGTAACC |
|  | Hdtd-R | CAAACCCACTAAAAGCG |
| *BteSult* | eSult-F | TTCCGTGCCACCAAAC |
|  | eSult-R | ACGACACCAAAACATCC |
| *Bttrypsin-1* | trypsin-1-F | GTTCCCGTGGTTGGTTTC |
|  | trypsin-1-R | TCATCGTCGCAGTTGCC |
| *BtCp7* | Cp7-F | AATCCCGCAACGGTCAC |
|  | Cp7-R | GGCTGGGGCAACAACTG |
| *BtERD6-7* | ERD6-7-F | ATCGGACCCATCTTCATC |
|  | ERD6-7-R | AGCACTTCTCGGCTTTTT |
| *BtHsp68* | Hsp68-F | CAAACATTTACAACCTACGC |
|  | Hsp68-R | CTTCTCTTGACAATCTACCC |
| *BtHsp70* | Hsp70-F | AGGGCTGAATGTTTTGC |
|  | Hsp70-R | GTGCTCTGGGGTTTGTT |
| *BtPLA2G15* | PLA2G15-F | ATTGGGGAGACTGGGTA |
|  | PLA2G15-R | GCCTGAGGTTTTTGGTT |
| *BtGLO4* | GLO4-F | GGAATCCTGACCAGAGAA |
|  | GLO4-R | GGACGCCCAATAAAAAC |
| *BtP450* | P450-F | GGAGGAAGTGACAGACC |
|  | P450-R | GTAAAAACGAATACGGG |
| *BtCar3* | Car3-F | GCGATGCGGTAGAGTTG |
|  | Car3-R | CGAAAGGTTTGCTGAAGA |
| *BtPAP* | PAP-F | CTCCGAATGGCGATAAT |
|  | PAP-R | AAACCCGTCCAAAGATA |
| *BtGld* | Gld-F | GCAGACTTCACGCCTCACCT |
|  | Gld-R | CCCAAGCCTTTGCCCATA |
| *BtCp* | Cp-F | GTCGTAGGTTTTTTGCTCT |
|  | Cp-R | ACCCTTATTCTCCTCGTTA |
| *BtMal-A1* | Mal-A1-F | CTACCCGCAATCCTTCT |
|  | Mal-A1-R | TGGTGCCGTATCTTTTATC |
| *BtTret1* | Tret1-F | ATTGTTGGCTTCGGACG |
|  | Tret1-R | CGATAAAATACCCAGACTTGCT |
| *BtAcads* | Acads-F | ATCATCCCTATTCCGACT |
|  | Acads-R | CACCTTCTTTACCAACCA |
| *BtLip* | Lip-F | AAGCAAATGGACGAGAGC |
|  | Lip-R | TTGATTAGATTCGGCAGAC |
| *BtCHI3* | CHI3-F | GGAAAGACAGACCCCGA |
|  | CHI3-R | TGCCGTCAACACAGTAATAGA |
| *BteIF5B* | eIF5B-F | TGTGCGGGTGCGGTTTA |
|  | eIF5B-R | TGGGAGTTCTCTCGGGTGTC |
| *BtFAAH2* | FAAH2-F | TCAACAACGACACACACATC |
|  | FAAH2-R | ACATCGCAAAAAGCACG |
| *BtTo* | To-F | GCCGAGACATCACACAA |
|  | To-R | GGGATACCCTTAGCCAA |
| *BtYWH* | YWH-F | CGCTGCGAGTGAGAGTAA |
|  | YWH-R | GAGGGTGCGAATGAGGT |
| *BtUgt1a8* | Ugt1a8-F | CTCCAAATGTGCTCCTGC |
|  | Ugt1a8-R | CCCTATCAACCGTTTCTACC |
| *BtCP21* | CP21-F | GACGCCAACCCCCAATACA |
|  | CP21-R | CAAGCAAGCAGCGAAGATGATA |
| *BtIdh3g* | Idh3g-F | GTCTTCTTGGAGGTGCT |
|  | Idh3g-R | GTTCTTCCCTGCGATTG |
| *BtY82E* | Y82E-F | CCCGAACCTCCCTTATC |
|  | Y82E.3-R | ATCCAGCCACTCCAACA |
| *BtElovl7* | Elovl7-F | CTGGATGTGCGAACCTA |
|  | Elovl7-R | CCAACCATTCCTGAGTG |
| *BtSnt* | Snt-F | TTACCCCAAATGGCAAC |
|  | Snt-R | GCATAAGACAAATGAACGC |
| *BtEF1-α* | EF1-α-F | TAGCCTTGTGCCAATTTCCG |
|  | EF1-α-R | CCTTCAGCATTACCGTCC |

**Supplementary Table S2.** UID deduplication basic data statistics for RNA-seq.

| **Sample** | **Q20(%)** | **Q30(%)** | **Deduplicated_Percentage** | | **GC (%)** | **clean_read** | **clean2raw_read_ratio (%)** | **raw_base**  **(G)** | **clean_base (G)** | | **clean2raw_base_ratio (%)** | |
| --- | --- | --- | --- | --- | --- | --- | --- | --- | --- | --- | --- | --- |
| B21_rep1 | 100 | 99.15 | | 96.55 | 44 | 47338580 | 73.45 | 9.67 | | 3.33 | | 34.44 |
| B21_rep2 | 100 | 99.20 | | 97.35 | 46 | 30317220 | 64.60 | 7.04 | | 1.97 | | 27.98 |
| B21_rep3 | 100 | 99.15 | | 93.65 | 42 | 76128992 | 76.25 | 14.98 | | 6.10 | | 40.72 |
| B26_rep1 | 100 | 99.15 | | 94.90 | 43 | 68580966 | 76.10 | 13.52 | | 5.28 | | 39.05 |
| B26_rep2 | 100 | 99.15 | | 94.90 | 43 | 65373972 | 75.62 | 12.97 | | 5.08 | | 39.17 |
| B26_rep3 | 100 | 99.15 | | 93.70 | 43 | 67742342 | 77.30 | 13.15 | | 5.66 | | 43.04 |
| B31_rep1 | 100 | 99.20 | | 97.30 | 45 | 41408302 | 75.21 | 8.26 | | 2.80 | | 33.90 |
| B31_rep2 | 100 | 99.15 | | 96.85 | 45 | 45332760 | 75.94 | 8.95 | | 3.16 | | 35.31 |
| B31_rep3 | 100 | 99.15 | | 96.15 | 44 | 67816280 | 76.22 | 13.35 | | 4.94 | | 37.00 |

**Supplementary Table S3.** Assay for Transposase-Accessible Chromatin with high-throughput sequencing (ATAC-seq) data quality control statistics.

| Sample | Clean Reads | Clean Unique Ratio (%) | Reads with UIDs | Consensus Reads | Dedup Reads | Dedup Unique Ratio (%) | Clean Bases | Clean GC (%) | Dedup Bases | Dedup GC (%) |
| --- | --- | --- | --- | --- | --- | --- | --- | --- | --- | --- |
| B21_rep1 | 42166888 | 54.07 | 40375886 (95.75%) | 37216328 (88.26%) | 37203026 (88.23%) | 75.13 | 5947881144 | 44.322 | 4793152950 (80.59%) | 43.203 |
| B21_rep2 | 49433090 | 51.18 | 47299436 (95.68%) | 43549938 (88.1%) | 43522988 (88.04%) | 72.15 | 7011109949 | 43.477 | 5643310171 (80.49%) | 42.277 |
| B21_rep3 | 41474828 | 52.06 | 39695518 (95.71%) | 36578668 (88.19%) | 36564936 (88.16%) | 73.20 | 5876256626 | 43.964 | 4733360108 (80.55%) | 42.798 |
| B26_rep1 | 41311134 | 54.27 | 39550740 (95.74%) | 36693100 (88.82%) | 36679284 (88.79%) | 74.70 | 5802178311 | 44.583 | 4704454118 (81.08%) | 43.475 |
| B26_rep2 | 40256766 | 51.77 | 38538948 (95.73%) | 35440672 (88.04%) | 35426910 (88%) | 73.59 | 5721791450 | 43.882 | 4601861351 (80.43%) | 42.707 |
| B26_rep3 | 44492178 | 55.90 | 42574146 (95.69%) | 39470788 (88.71%) | 39457314 (88.68%) | 76.42 | 6127721342 | 44.790 | 4952049439 (80.81%) | 43.682 |
| B31_rep1 | 37161626 | 55.01 | 35560986 (95.69%) | 32908764 (88.56%) | 32899964 (88.53%) | 76.24 | 5203419493 | 45.076 | 4204582918 (80.8%) | 44.006 |
| B31_rep2 | 46846728 | 52.48 | 44797422 (95.63%) | 41272328 (88.1%) | 41261408 (88.08%) | 73.68 | 6662115826 | 45.619 | 5362850987 (80.5%) | 44.620 |
| B31_rep3 | 37452018 | 50.89 | 35805966 (95.6%) | 32835634 (87.67%) | 32825356 (87.65%) | 72.82 | 5395934070 | 44.775 | 4327755774 (80.2%) | 43.705 |

**Supplementary Figure S1.** The Cluster heatmap formed by differentially expressed genes.
